# Contemporary Developments in the Cambridge Study in Delinquent Development: David Farrington's Legacy

**DOI:** 10.1002/cbm.2381

**Published:** 2025-03-23

**Authors:** Darrick Jolliffe, Hannah Gaffney, Manuel Eisner, David P. Farrington

**Affiliations:** ^1^ Department of Law and Criminology Royal Holloway University of London Egham UK; ^2^ School of Law and Criminology Ringgold Standard Institution University of Greenwich London UK; ^3^ Institute of Criminology Ringgold Standard Institution University of Cambridge Cambridge UK

## Abstract

**Background:**

The Cambridge Study in Delinquent Development (CSDD) is one of the most important prospective criminological longitudinal studies in the world. This now‐famous study of 411 boys, followed up from age 8 (in 1961) to 48 with in‐person social interviews and up to 61 in official records, has produced an immense range and depth of knowledge.

**Aims:**

The aim of the current paper is to describe recent efforts that have been made to both secure the data available from the CSDD by digitising the historical paper records and to obtain new data by undertaking a new wave of data collection with the men at about age 70.

**Results:**

Both the archiving of paper records and the new interviews significantly expand the depth and range of research questions that the CSDD can address. A surprisingly high proportion of the selected samples of men interviewed at about age 70 self‐reported an offence.

**Conclusions:**

These new developments solidify the status of the CSDD, David Farrington's legacy, as a world‐leading source of information about the development and maintenance of criminal and antisocial behaviour.

## 1 Introduction

The Cambridge Study in Delinquent Development (CSDD) is one of the longest and most rigorous prospective longitudinal studies in the world, focusing on the development, maintenance and discontinuity of offending/antisocial behaviour and the factors associated with these outcomes. To date, this study has produced 323 publications (including this one), but others are known to be at various stages of production. The CSDD is almost peerless in the comprehensiveness of the data that have been collected. This includes the number of assessments that have been undertaken, the range of data sources and the fact that the length of follow‐up is one of the longest of any longitudinal study in criminology. The CSDD therefore provides some of the most complete knowledge about offending and other associated outcomes across the life course.

The CSDD is a prospective longitudinal survey of 411 London males who were first studied in 1961–1962 at ages 8–9. Their parents, teachers, peers, female partners and children have also been interviewed. In the interests of clarity, the original 411 males are called generation 2 (G2), their parents are called generation 1 (G1) and their children are called generation 3 (G3). However, this paper focuses on the original cohort of G2 males, and so they will be referred to as the males.

The CSDD was initiated by Professor Donald West (who sadly died in 2020, aged 95) in 1961. Professor David P. Farrington (who recently passed away in November 2024, aged 80) joined Professor West in 1969 and took over the direction of the CSDD in 1982. The age 48 interviews were carried out in collaboration with Professor Jeremy Coid, and Professor Darrick Jolliffe joined the CSDD as co‐director in 2020.

Remarkably, a prospective longitudinal study was neither Donald West's first (nor even second) choice for his research focus in his new role as assistant director of research at the Institute of Criminology, University of Cambridge nor was it the Director's choice (Sir Leon Radzinowicz; see Farrington et al. 2021). However, then as now, academics were beholden to funders, and Donald's idea for a prospective longitudinal study was considered attractive to the Home Office, which funded this. Over the years, the Home Office and the Department of Health have been the main funders of the CSDD with contributions from the Department for Education, the Rayne Foundation, the Barrow Cadbury Trust and the Smith Richardson Foundation.

At the time they were first contacted in 1961–1962, the males were all living in a working‐class area of South London (which is never identified). The sample was chosen by taking all the males who were then aged 8–9 and were on the registers of six state primary schools, within a 1‐mile radius of a research office, which had been established. In addition to 399 males from these six schools, 12 males from a local school for ‘educationally subnormal’ (special needs) children were included in the sample. This was an attempt to make it more representative of the population of males living in the area. Therefore, the CSDD males were not a probability sample drawn from a population but rather a complete population of males of that age in that area at that time.

In nearly all cases (94%), their family breadwinner in 1961–1962 (usually the father) had a working‐class occupation (skilled, semi‐skilled or unskilled manual worker). Most of the boys (357 or 87%) were White and British. Of the remainder, 14 (3.4%) had at least one parent of West Indian or African origin, 12 (3%) had at least one parent from Cyprus and 16 (4%) had at least one parent from another country. The majority of the males were living in conventional two‐parent families with both a father and a mother figure; at age 8, only 6% of the males had no operative father and only 1% had no operative mother.

The original aims of the CSDD were to describe the development of delinquent and criminal behaviour in inner‐city males, to investigate to what extent this could be predicted in advance and to explain why juvenile delinquency began, why it did or did not continue into adult crime and why adult crime often ended as men reached their twenties. The main focus was to study continuity or discontinuity in offending behaviour and the effects of life events on development. The CSDD was not designed to test any one particular theory about delinquency but aimed to test many different hypotheses about the causes and correlates of offending. However, an influential theory (the Integrated Cognitive Antisocial Potential theory ‘ICAP’) was later proposed to explain its findings (Farrington 2005, 2020c). The results of the CSDD have been described in seven books (Farrington et al. 2023; Farrington et al. 2013; A. R. Piquero et al. 2007; West 1969, 1982; West and Farrington 1973, 1977) and in seven summary articles (Farrington and West 1981, 1990; Farrington 1995, 2003, 2019, 2021; Farrington et al. 2009).

The males were interviewed nine times, at ages 8, 10, 14, 16, 18, 21, 25, 32 and 48. At all ages except 21 and 25, the aim was to interview all the males who were still alive. Because of inadequate funding, only about half of the males were interviewed at age 21 and about a quarter at age 25. At age 21, the aim was to interview all the convicted males and an equal number of randomly chosen unconvicted males, and 218 of the 241 target males (90%) were interviewed. At age 25, only 85 males were interviewed.

In the other interviews, it was always possible to interview a high proportion of the males who were still alive: 405 (99%) at age 14, 399 (97%) at age 16, 389 (95%) at age 18, 378 (94%) at age 32 and 365 (93%) at age 48. At age 48, 17 males had died (of whom 13 were convicted), five could not be traced and 24 refused, which meant that 365 out of 394 (93%) who were alive were interviewed. The males also completed a medical interview at age 48. Out of 343 who completed the age 48 social interview face to face in person, 304 (89%) completed the medical interview. At age 32, 289 of the 378 interviewed G2 males (76%) were living with a wife or female cohabitee, and 268 of these G2 females (93%) filled in a questionnaire about child rearing. At age 48, 299 of the 365 interviewed G2 males (82%) were living with a female partner, and 254 of these G2 females (85%) were interviewed.

Farrington et al. (2023) highlighted the main contributions of the CSDD in the last 10 years (2013–2023). These included examining the childhood risk factors (measured at ages 8–10) of chronic offenders (i.e., those with 5 or more convictions from ages 10 to 61) and life course persistent offenders (LCP, i.e., those who had a criminal career duration of at least 20 years between their most recent offence and age 61), and illustrating that the childhood predictors of convictions did not overlap with those of criminal career duration (Farrington 2020a). Therefore, the considerable knowledge about the risk factors for offending identified in much past research may not be applicable to understanding LCP offending. Other key contributions include the identification of the important interactions between low‐resting heart rate and childhood risk factors that have an impact on later offending (Farrington 2020b), the development of psychopathy (Farrington and Bergstrom 2022), the overlap between criminal violence and intimate partner violence (IPV, A. Piquero et al. 2014) and the intergenerational transmission of crime (Farrington et al. 2017), imprisonment (Auty et al. 2022), adverse childhood experiences (Craig et al. 2021), mental health (Reising et al. 2019) and IPV (Shakoor et al. 2022). The CSDD has also been used to highlight the link between an antisocial lifestyle and negative health outcomes (Skinner and Farrington 2021), the importance of certain life events on offending (i.e., the birth of a first child; Theobald et al. 2015) and the monetary costs of official and self‐reported offending (Raffan Gowar and Farrington 2013).

## 2 Follow‐Up of CSDD Men at Age 70

It was an aim of David's to follow up the CSDD men with another social interview. This was in part because he expressed an interest to have a ‘completed’ longitudinal study, but also likely because of his long‐standing interest in criminal career duration and his oft‐articulated suspicion that in previous research on desistance, the termination of offending may have been overindicated. For example, Sampson and Laub (2003) searched the criminal and death records (up to age 61–70) of 475 of the 500 original male delinquents who had been included in Glueck's seminal prospective longitudinal study in Cambridge, Massachusetts. Most (57%) of the men who had survived up to age 50 had no further arrests after age 40, resulting in Sampson and Laub concluding that ‘Ageing out of crime is thus the norm—even the most serious delinquents desist’ (Sampson and Laub 2003, 569) and furthermore ‘all offenders desist but at time‐varying points across the life course’ (Sampson and Laub 2003, 588). They also dismissed the concept of ‘life course persistent’ offenders and further asserted that adult trajectories of offending could not be predicted from childhood (Sampson and Laub 2003, 584–585).

However, using the results of the age 61 criminal record searches of the CSDD, Farrington (2020a) showed that a notable proportion of the sample continued to be convicted. Over 8.4% of the 371 men still at risk between the ages of 50 and 61 had been convicted, and these individuals had an average of about 1.9 convictions. It is important to note that these were not all convictions for trivial offences. Over 55% of the 58 convictions accrued between the ages of 50 and 61 were for assault, threats and sex offences. In addition, two men had their first conviction in this age range. Collectively, this suggests that official offending at later ages may be more common than previously identified and that studies which do not have long follow‐ups may miss this.

It makes intuitive sense that, with a longer follow‐up and therefore a greater opportunity for offences to be committed and identified, more accurate assessments of who has actually stopped offending can be made. It would appear that much of the previous research on desistence, as well as other important criminal career parameters such as escalation (i.e., increasing seriousness of offending) and intermittency (i.e., time intervals between two criminal careers when the underlying rate of offending is zero; Farrington et al. 2020), may be based on incomplete data. Similarly, although some research has examined the childhood risk factors of criminal career duration (e.g., Jolliffe et al. 2017), the existence and/or the strength of these relationships would be influenced by more accurate assessments of who has or has not continued offending.

Another important limitation of much criminal career duration research is that this has mostly been based on official records of offending (as described by Farrington 2020a). Because of the well‐known limitations of official records, particularly in terms of missing a considerable amount of offending behaviour (e.g., Farrington et al. 2003) and potential bias in terms of who receives an official offence, it is important to study criminal career duration using self‐reports obtained from repeated personal interviews. As part of the social interviews of the CSDD, the males answer questions about their self‐reported offending using a card sorting method. This involves having the participants’ place cards containing descriptions of offences into sealed envelopes which the interviewer opens and codes later, making the reporting of these behaviours confidential.

Attempts to obtain sufficient funding to follow up all the CSDD men began in 2013 and continue to the present. Unfortunately, to date, these attempts have not proven successful. Generally, the feedback received from funding agencies has identified a number of positive reasons for considering funding the follow‐up of the CSDD men. These include the very low attrition of the study (which increases the validity of the conclusions that can be drawn [e.g., West and Farrington 1977]), the comprehensive data obtained from multiple sources based on repeated measures and the fact that this means the impact of changes in risk and protective factors on outcomes in old age could be reliably assessed.

The main limitations that have been noted by funders include the fact that the CSDD did not have a specific theory to test and that this was a relatively small sample that is quite homogeneous (i.e., all male, mostly White, from one deprived London neighbourhood). This was viewed to make the CSDD of limited contemporary policy relevance.

Of course, we disagree with these negative assessments and believe that following up the CSDD cohort would significantly add to our knowledge about criminal career duration and extend what we understand about successful ageing (i.e., extending Farrington et al. 2006). In fact, the CSDD would be uniquely placed to test important sociological theories of ageing, such as continuity theory, which proposes that as people age, they try to maintain or continue previous habits, preferences, commitments, values, beliefs and all the factors that have contributed to their personalities (Atchley 1989). In addition, a full follow‐up of the men at age 70 would provide essential data on criminal career duration to test the assumption of Sampson and Laub (2003) that factors in childhood do not have an impact on later life.

Additionally, although the sample size could be considered small, the statistical power of any analysis depends both on the sample size and on the effect size of any comparison, which may be unknown. Regardless, the measures typically reported for CSDD outcomes are measures of effect (odds ratios), which are less influenced by sample sizes. The ‘small’ sample size also makes it possible to capture in‐depth qualitative narratives, for example, about the details of fights that occurred when the males were 18 years old (Farrington et al. 1982) and comprehensive life histories (e.g., Zara and Farrington 2015).

With a shoestring budget of underspent funds from other grants, attempts to follow up a subsample of the CSDD men began in earnest in late 2019. This started with minor editing of the age 48 social interview schedule to ensure that the wording of all questions was sufficiently contemporary. Additionally, some new questionnaires were included (e.g., the Basic Empathy Scale; Jolliffe and Farrington 2006), as were questions about the quality of relationships with family members, including grandchildren. It is possible that relationships with grandchildren may increase or decrease the well‐being and health of the CSDD male (grandparent; Leimer and Van Ewijk 2022). Ethical clearance was obtained, and data sharing agreements were finalised. Very fortunately, one of the highly experienced interviewers from the age 48 interviews (and children’s interviews, G3) agreed to come on board to support this study. Along with Professor Georgia Zara, a list of 50 men was drawn up based on those who were thought to be alive and an assessment of their criminal career duration up to age 61 in official records.

After a significant delay because of COVID‐19, the first interview was conducted in late 2021, and to date, 30 full social interviews with the CSDD men have been completed. The average age of the 30 men was 69.6 (SD = 0.99); half the men lived with wives, 20% lived with partners and 30% lived alone. Two‐thirds of the men reported that their health did not limit their day‐to‐day lives, and over 70% reported that their health was good, very good or excellent, whereas over 40% reported going out once or more per week. Quite surprisingly, considering the humble beginnings of the CSDD men, 70% reported that their childhood was happy or very happy. Additionally, surprising was that 36.7% of the males self‐reported an offence within the last 5 years. This included kicking, punching or threatening and serious drug use.

Of course, these results should be considered exploratory. With limited funds to follow up on the men, we targeted those suspected of living in and around London, which may have resulted in the sample being skewed.

### 2.1 Archiving the Paper Records

Obviously, the CSDD began well before computers and digital data storage were available, and although much of the quantitative data has been computerised for analyses, a considerable amount of paper records has been generated as part of this study. This included paper records from the 1960s and 1970s, such as the original paper interview schedules and the notes from the psychiatric social workers about their impressions of the family and the boys. In fact, paper records continued to be created even into the early 2000s, with the ‘preinterview sheets’, which contain specific details of every attempt that the interviewers made to contact the male in the lead up to a successful (or unsuccessful) interview. These preinterview sheets have been used to produce papers about tracing the men (e.g., Farrington 2015; Farrington et al. 1990).

These paper records also included qualitative data that had been collected in the many interviews but had rarely been analysed. For example, at age 18, the males were asked to describe their recent fights (i.e., in the last year), their use of weapons in these fights, their fighting at football matches, and they were prompted to discuss their interactions with the police in the last year.

For example, Participant 1013 states:

I've had a punch‐up and that. It's never been a proper fight like when I got beaten up anything.

The participant had never used a weapon in a fight but states:

‘I wouldn't do, though like maybe if I was getting beaten up in a fight’. I've had like knives on me, or steel combs when the teeth are sharpened and takes some of the teeth out, like flash, but I don't think I'd use it unless I was getting really beaten up or they touched me face like.

The participant reported being pushed about a lot by police in various incidents and searched several times but was never hit or beaten by the police in the previous 3 years. He has been searched about 15 times in the last 12 months.

’Cause they (police) know us—always pick on us. Cause I've never been away—whenever I've been to court I've never been sent away except on remand. They (police) want to get their own back’. If it ain't like me who's done it, they say it is, you know what I mean—stop and search me.

In addition to specific qualitative data, such as the descriptions of fights or the assessment notes of the psychiatric social workers, the thoroughness and range of sources included in the paper records of the CSDD mean that these can be used to produce detailed life histories of the men. For example, Zara and Farrington (2015) used the paper records from the CSDD to construct thorough life histories of 8 men who had long criminal careers. These case histories included in‐depth detail about the background of the males' parents (G1), the males' early relationships with their siblings, friends and schools and their later relationships with employers and partners. These case histories provide helpful contextual insight into the males' persistent involvement in criminal and antisocial behaviour and illustrate how cumulative exposure to multiple risk factors was experienced depending on certain social or family backgrounds, peer networks or individual characteristics.

A total of 16 filing cabinets of paper records of CSDD data, tests and associated material had been collected over the years. Each filing cabinet contained different tests or data, but these had been meticulously organised by the main participant ID. These cabinets had been moved from the Institute of Criminology, University of Cambridge to Queen Mary University of London for the age 48 interviews in the early 2000s and then returned to the Institute in the late 2000s upon completion of the G3 interviews (i.e., the interviews of the children (G3) of the original males; see Farrington et al. 2021).

A lack of office space in which to have 16 filing cabinets accessible prompted David Farrington to move these to a residential building in about 2014, shortly after his official ‘retirement’ from the Institute of Criminology. David knew these files contained irreplaceable information about the CSDD, and he wanted them kept accessible so that they could be used to continue to generate knowledge such as that described above (i.e., Zara and Farrington 2015).

However, increased institutional concern about data security and an awareness that the paper records would eventually deteriorate led to efforts to electronically scan all the paper records. There is considerable literature detailing the important benefits and best approaches to digital data archiving (Doorn and Tjalsma 2007). These include the potential to maximise data use and avoid so‐called data waste while providing new ways to examine and search the data (e.g., James and Sørensen 2000). This democratisation of data offers users an increased opportunity to interact with the data in a nonlinear format and adopt constructivist approaches to conducting their research (Bolick 2006). Digital archives such as those of the CSDD records also provide the opportunity to make use of developments in secondary qualitative data analysis (e.g., Heaton 2008).

Professor Manuel Eisner and Professor Darrick Jolliffe received a grant to digitise the paper records from the Newton Fund, and this study was led by Dr Hannah Gaffney. This involved creating an inventory of all the paper records, managing a private scanning company to attend the residence and collect the records, quality‐assuring the scans that were produced, ensuring their secure storage within the Institute of Criminology and taking steps to organise and inventory this data. This was an enormous undertaking, and Dr Gaffney did a fantastic job. What was once 16 filing cabinets is now stored on one secure computer (not connected to the internet). There is still considerable work to be done in cataloguing and organising these data, and the process for access for future research is still being established but collaborations with those interested in using these data will soon be welcomed.

## 3 Conclusion

David Farrington was such a prolific academic that a case could be made for him having a legacy in experimental criminology, school bullying and evidence synthesis among others, but we would argue that David's name will always be synonymous with developmental criminology and the CSDD. By digitising the paper records and commencing the interviews of the men at age 70, steps have been taken to solidify and extend David's legacy. We will continue to search for funding to interview all the men and then, as David urged, push on to try to reinterview the children (G3) and perhaps, as David also suggested, interview the grandchildren (G4). David was always supportive of fruitful collaborations, and we hope that researchers can pose important and innovative research questions that can be tested using the CSDD so that this important study can continue to push forward (or back?) the boundaries of knowledge.

## Conflicts of Interest

The authors declare no conflicts of interest.

## Data Availability

The authors have nothing to report.
